# Administration of Methylene Blue to Facilitate the Management of Post-Cardiac Arrest Refractory Shock

**DOI:** 10.1016/j.jaccas.2026.109654

**Published:** 2026-08-06

**Authors:** Sagar Rastogi, Maxwell Droznin, Rachael Steinhauer, Evans Osuji, Farion Cooper, Sammy Zakaria

**Affiliations:** a Johns Hopkins University, Baltimore, Maryland, USA; b Department of Medicine, Johns Hopkins University School of Medicine, Baltimore, Maryland, USA; c Division of Cardiology, Department of Medicine, Johns Hopkins University School of Medicine, Baltimore, Maryland, USA

**Keywords:** acid-base imbalance, hemodynamics, perfusion, treatment, ventricular fibrillation

## Abstract

**Background:**

Post–cardiac arrest refractory shock is associated with over 50% mortality, with limited evidence-based treatment options. Methylene blue (MB) has shown promise for post–cardiopulmonary bypass vasoplegia, but its use after cardiac arrest outside the cardiac surgery setting remains unexplored.

**Case Summary:**

A 50-year-old man with recurrent hypothermia-associated ventricular fibrillation arrests presented with refractory vasoplegic shock requiring maximum institutional doses of norepinephrine (3.0 μg/min), vasopressin (0.06 units/min), and epinephrine (1.0 μg/kg/min). Following administration of a 2 mg/kg MB bolus and 0.5 mg/kg/h continuous infusion, the patient demonstrated rapid hemodynamic stabilization with vasopressor down-titration, lactate clearance (20-7.6 mmol/L), and acidosis correction (pH 6.93-7.4) within 11 hours. The patient ultimately died from multiorgan failure.

**Discussion:**

This novel human case suggests MB may facilitate hemodynamic stabilization in post–cardiac arrest refractory shock.

**Take-Home Message:**

Earlier MB administration may warrant consideration as rescue therapy in post–cardiac arrest refractory shock when conventional vasopressors are maximized.

## Visual Summary

**Figure:**
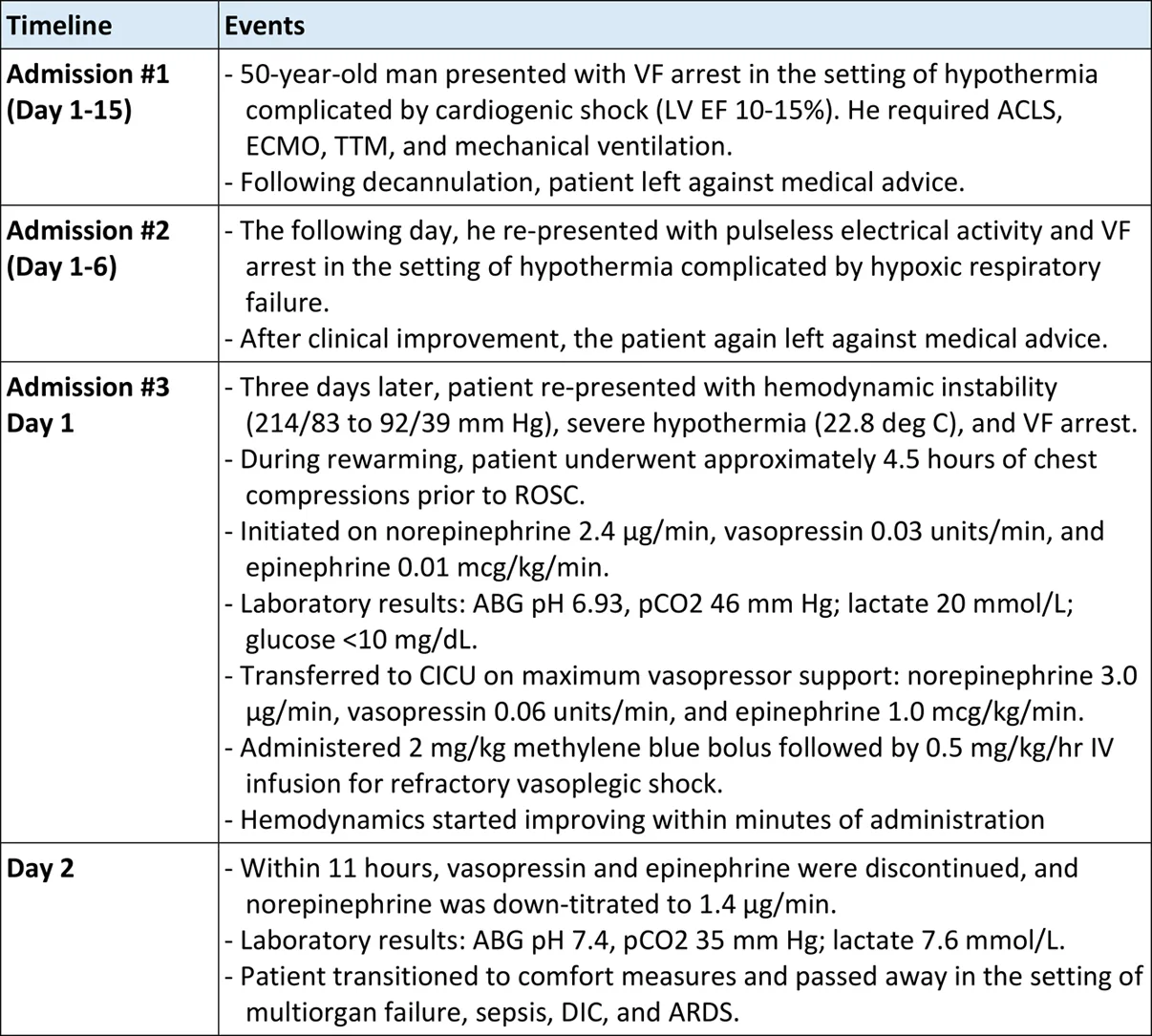

Postcardiac arrest shock is a complex hemodynamic state involving a combination of myocardial dysfunction and systemic ischemia–reperfusion injury.^1^ In order to maintain adequate tissue perfusion, approximately 75% of postcardiac arrest patients require vasopressor therapy.^2^ In more severe situations, patients develop refractory vasoplegia, characterized by persistent hypotension, metabolic acidosis, and progressive end-organ hypoperfusion, despite attempts at fluid resuscitation and being loaded with high-dose vasopressors, oxygenation, and ventilation.^3^

Methylene blue (MB) is a medication approved by the Food and Drug Administration to treat methemoglobinemia. While off-label, its use has expanded to patients in vasopressor-resistant septic shock or with post–cardiopulmonary bypass vasoplegia; however, there are no reports for use in post–cardiac arrest cardiogenic or vasoplegic shock states, indicating limited evidence to guide clinical practice.^4^

The therapeutic effects of MB are mediated through both its redox activity and modulation of the nitric oxide–cyclic guanosine monophosphate (NO-cGMP) pathway. As a reducing agent, MB accelerates the conversion of methemoglobin to hemoglobin, thereby improving oxygen-carrying capacity and tissue perfusion.^5^ The etiology of refractory shock is described as a lack of response to catecholamines with an upregulation of inducible nitric oxide synthase (NOS) leading to excessive production of NO.^3^ NO diffuses into vascular smooth muscle cells to activate soluble guanylate cyclase, increasing intracellular cGMP. On the cellular level, the increase of cGMP leads to relaxation of smooth muscle cells, which causes vasodilation and clinically presents as persistent hypotension. By diffusing into cells and inhibiting NOS and the activity of guanylate cyclase, MB reduces cGMP production and restores vascular tone. MB not only increases systemic vascular resistance and mean arterial pressure (MAP), but it also improves vascular response to standard catecholamines.^5^

Building on 1 recent report successfully using MB following out-of-hospital cardiac arrest, we report another use of MB to manage refractory vasoplegic shock following cardiac arrest.^6^ The patient, who experienced 3 hypothermia-associated cardiac arrests within 1 month, demonstrated a rapid hemodynamic response to MB after failing to respond to maximum doses of 3 other conventional vasopressors.

## Case Presentation

### History of presentation

A 50-year-old man with a history of polysubstance use disorder (alcohol, cocaine, and cannabis) presented to the emergency department with ventricular fibrillation (VF) arrest in the setting of severe hypothermia (22.8 °C). He had 2 recent hospitalizations for VF arrest related to hypothermia: the first requiring advanced cardiac life support, venoarterial extracorporeal membrane oxygenation (ECMO), targeted temperature management, and mechanical ventilation for cardiogenic shock with severe biventricular failure (ejection fraction: 10%-15%); the second presenting with pulseless electrical activity and VF arrest with acute hypoxic respiratory failure. Following both prior admissions, the patient left against medical advice.

On the current presentation, cardiopulmonary resuscitation was initiated and maintained using a LUCAS device (Stryker), with approximately 4.5 hours of compressions required before return of spontaneous circulation because of prolonged rewarming. Initial blood pressure was 214/83 mm Hg, trending down to 92/39 mm Hg.

### Past medical history

The patient's past medical history was notable for polysubstance use disorder including alcohol, cocaine, and cannabis use. He had 2 recent hospitalizations within a month of this presentation for VF arrest in the setting of hypothermia, with the initial hospitalization requiring ECMO support for cardiogenic shock.

### Differential diagnosis

Given the clinical presentation of refractory hypotension despite vasopressor therapy, the differential diagnosis included cardiogenic shock, distributive (vasoplegic) shock, mixed shock physiology, septic shock, and adrenal insufficiency. The persistent hypotension despite maximum vasopressor doses, along with the severe metabolic acidosis and elevated lactate, suggested a significant vasoplegic component contributing to the shock state.

### Investigations

Arterial blood gas revealed severe acidemia (pH 6.93, pCO_2_ 46 mm Hg), lactate trending to 20 mmol/L, and hypoglycemia (glucose <10 mg/dL). Coagulation studies demonstrated disseminated intravascular coagulation (DIC) with international normalized ratio 2.85 and fibrinogen <42 mg/dL. The clinical picture was further complicated by acute respiratory distress syndrome (ARDS).

### Management

Initial management included vasopressor support with norepinephrine 2.4 μg/min, vasopressin 0.03 units/min, and epinephrine 0.01 μg/kg/min. Despite escalation to maximum institutional doses (norepinephrine 3.0 μg/min, vasopressin 0.06 units/min, epinephrine 1.0 μg/kg/min), the patient's MAP remained <65 mm Hg.

Given concern for a vasoplegic component contributing to persistent hypotension, MB was administered as a 2 mg/kg bolus followed by 0.5 mg/kg/h continuous intravenous infusion. Within minutes of MB administration, hemodynamic improvement was observed.

### Outcome and follow-up

Within 11 hours of MB administration, the patient demonstrated notable hemodynamic stability, with down-titration of norepinephrine to 1.4 μg/min and discontinuation of vasopressin and epinephrine. Lactate decreased from 20 to 7.6 mmol/L, and acidosis improved (pH 7.4, pCO_2_ 35 mm Hg).

Despite hemodynamic improvement, the patient remained critically ill with multiorgan failure, sepsis, DIC, and ARDS. Given his poor prognosis, goals of care were transitioned to comfort-only measures, and the patient subsequently passed away.

## Discussion

This case highlights the potential benefit of prompt administration of MB in patients with postarrest refractory shock associated with severe hypothermia. Despite following 2025 American Heart Association post–cardiac arrest guidelines,^7^ the patient remained critically hypotensive in a state of refractory shock. He was one of the 6% to 7% of intensive care unit patients who developed refractory shock postarrest, with an associated mortality exceeding 50%. Current American Heart Association guidelines recommend norepinephrine as the suggested first-line vasopressor, but acknowledge the lack of randomized controlled trials in this population.^7^

The existing literature on MB in shock is concentrated in 2 clinical domains: post–cardiopulmonary bypass vasoplegia and septic shock. A systematic review and meta-analysis of 15 studies including 832 patients demonstrated that MB coadministered with vasopressors reduced mortality (OR: 0.54, 95% CI: 0.34-0.85) and decreased vasopressor requirements in vasodilatory shock.^8^ However, a meta-analysis of 6 randomized controlled trials found that MB did not significantly reduce mortality (OR: 0.59, 95% CI: 0.32-1.06), although it shortened intensive care unit and hospital length of stay.^9^ Critically, the most current systematic review on MB in cardiogenic shock and cardiac arrest (Batchelor et al,^4^ 2025) screened 676 records across 5 databases and identified no published human case reports in this context, with only 6 preclinical animal studies and 1 retrospective cohort study without detailed postarrest outcome data. Preclinical animal studies have shown mixed results regarding MB efficacy in cardiac arrest models, underscoring the importance of clinical case reports to inform whether hemodynamic benefits translate to human patients.

The dosing regimen used in this case, a 2 mg/kg MB bolus followed by 0.5 mg/kg/h continuous infusion, is consistent with regimens associated with the best outcomes in the broader shock literature. Sari-Yavuz et al^10^ (2022) found in a retrospective cohort of 209 critically ill patients that a bolus followed by continuous infusion was associated with reduced 28-day mortality compared to bolus-only or infusion-only strategies, and that cardiogenic shock and higher norepinephrine doses increased the likelihood of responding to MB.

Despite maintaining a MAP of <65 mm Hg while on institution-maximum doses of norepinephrine, vasopressin, and epinephrine, our patient's refractory shock began to improve within 2 minutes after MB initiation. By 11 hours, norepinephrine was reduced from 3.0 to 1.4 μg/min, vasopressin and epinephrine were discontinued, lactate declined from 20 to 7.6 mmol/L, and pH normalized from 6.93 to 7.4. This temporal association between MB administration and hemodynamic stabilization is consistent with the mechanism of NO-cGMP pathway inhibition and restoration of vascular tone. Although the patient ultimately died from multiorgan failure, the hemodynamic response to MB was notable and temporally distinct from other interventions. Causality cannot be established from a single case, and the contribution of concurrent therapies cannot be excluded. However, the rapid improvement in vasopressor requirements and metabolic parameters after MB administration suggests that MB may warrant consideration as a rescue therapy in post–cardiac arrest refractory vasoplegic shock, particularly when conventional vasopressors have been maximized. Future research should consider exploring the potential benefit of earlier administration of MB in humans, before the development of irreversible multiorgan failure and as opposed to prolonged vasopressor therapy.

## Conclusions

This case represents a novel report of MB administration for refractory vasoplegic shock after cardiac arrest outside the cardiac surgery setting. The temporal association between MB administration and hemodynamic stabilization suggests MB may warrant consideration as rescue therapy when conventional vasopressors have been maximized. Future research should explore earlier MB administration before irreversible multiorgan failure develops.

**Take-Home Messages**

• Post-cardiac arrest refractory shock has over 50% mortality, with no evidence-based guidelines for effective treatment beyond standard vasopressors.
• Methylene blue (2 mg/kg bolus followed by 0.5 mg/kg/h infusion) was temporally associated with rapid hemodynamic stabilization, vasopressor down-titration, and lactate clearance in this case.
• Earlier MB administration may warrant consideration as rescue therapy in post–cardiac arrest refractory vasoplegic shock when conventional vasopressors are maximized.

## Funding Support and Author Disclosures

The authors have reported that they have no relationships relevant to the contents of this paper to disclose.

**Visual Summary:** Timeline of Key Clinical Events and Response After Methylene Blue Administration

Time	Events
Admission #1 (days 1-15)	A 50-year-old man presented with VF arrest in the setting of hypothermia complicated by cardiogenic shock (LVEF: 10%-15%). He required ACLS, ECMO, TTM, and mechanical ventilation. Following decannulation, patient left against medical advice.
Admission #2 (days 1-6)	The following day, he re-presented with pulseless electrical activity and VF arrest in the setting of hypothermia complicated by hypoxic respiratory failure. After clinical improvement, the patient again left against medical advice.
Admission #3 (day 1)	Three days later, patient re-presented with hemodynamic instability (214/83-92/39 mm Hg), severe hypothermia (22.8 °C), and VF arrest. During rewarming, patient underwent approximately 4.5 h of chest compressions before ROSC. Initiated on norepinephrine 2.4 μg/min, vasopressin 0.03 units/min, and epinephrine 0.01 μg/kg/min. Laboratory results: ABG, pH 6.93; pCO_2_, 46 mm Hg; lactate, 20 mmol/L; glucose, <10 mg/dL. Transferred to CICU on maximum vasopressor support: norepinephrine 3.0 μg/min, vasopressin 0.06 units/min, and epinephrine 1.0 μg/kg/min. Administered 2 mg/kg methylene blue bolus followed by 0.5 mg/kg/h IV infusion for refractory vasoplegic shock. Hemodynamics started improving within minutes of administration
Day 2	Within 11 h, vasopressin and epinephrine were discontinued, and norepinephrine was down-titrated to 1.4 μg/min. Laboratory results: ABG, pH 7.4; pCO_2_, 35 mm Hg; lactate, 7.6 mmol/L. Patient transitioned to comfort measures and passed away in the setting of multiorgan failure, sepsis, DIC, and ARDS.

ABG = arterial blood gas; ACLS = advanced cardiac life support; ARDS = acute respiratory distress syndrome; CICU = cardiac intensive care unit; DIC = disseminated intravascular coagulation; ECMO = extracorporeal membrane oxygenation; IV = intravenous; LVEF = left ventricular ejection fraction; ROSC = return of spontaneous circulation; TTM = targeted temperature management; VF = ventricular fibrillation.
